# A Case of Aseptic Loosening Secondary to Cement Debris

**DOI:** 10.7759/cureus.84955

**Published:** 2025-05-28

**Authors:** Dhivakaran Gengatharan, Haobin Chen, Joel Lim, Wei Ming Siow, Kein Boon Poon

**Affiliations:** 1 Orthopaedic Surgery, Sengkang General Hospital, Singapore, SGP

**Keywords:** aseptic loosening, cement, particulate wear, synovitis, total knee arthroplasty

## Abstract

Particulate wear is a well-established cause of aseptic loosening and failure in joint arthroplasty. We aim to describe a case of cement-induced synovitis with the deposition of cement debris causing a distinct green-hued synovitis in a case of primary total knee arthroplasty (TKA). A 61-year-old lady presented to our clinic with right knee pain and swelling after undergoing bilateral total knee replacement four years ago at another institution. She was initially worked up by her primary surgeon and was symptomatically treated with non-steroidal anti-inflammatory medication (NSAID). However, her symptoms persisted and impaired her daily function. She presented with right knee effusion and decreased range of motion of the knee, as well as joint line tenderness. Further radiological investigations showed right knee tibial component implant loosening, with bone cement fragments in the anterior tibia. As such, the patient was counselled and underwent a revision surgery. Intraoperatively, there was extensive synovitis with prominent green pigment deposits. Excessive cement was noted in the femoral notch, causing wear of the liner pole. The tibia tray was also found to be loose, with no cement adherent to the tibia tray, and all the cement mantle seated on the tibia bone. Postoperatively, the patient made an uneventful recovery. She was able to range her knee from 10-130 degrees with no pain within 2 months. Direct wear from residual cement and micromotion at the tibia tray due to poor cementing technique during the primary surgery were important factors leading to severe cement wear and failure. This clinical case is a reminder of the importance of good cementing technique to ensure a good outcome in TKA.

## Introduction

Total knee arthroplasty (TKA) is one of the most reliable and successful joint replacement procedures in orthopedic surgery, delivering excellent outcomes. Its popularity has grown significantly over the past decade, as it consistently provides effective pain relief and improved function for patients with arthritic knees. In the United States, the annual number of TKA procedures is projected to reach 1.26 million by 2030 [1]. However, with the ever-increasing adoption of TKA, the demand for revision surgeries has also risen steadily.

Aseptic loosening (AL) is the most common cause of revision TKA, accounting for 22.8% to 31.2% of cases [2,3]. AL is characterized by the gradual detachment of the implant from bone in the absence of infection and typically occurs at the implant-cement or bone-cement interface, with the latter being more common [4,5,6]. This complication is associated with patient dissatisfaction, increased morbidity, and higher healthcare costs due to the need for revision surgery [7,8]. Risk factors for AL include high body mass index (BMI), diabetes, implant malrotation or malalignment, and poor cementing technique [9,10].

The pathophysiology of AL remains poorly understood and a topic of ongoing debate. The prevailing theory suggests that debris particles from implant surfaces initiate an inflammatory cascade, disrupting bone homeostasis. Activated macrophages engulf wear particles, leading to osteolysis and eventual loosening of the prosthesis [11].

In this report, we present a compelling case of AL attributed to an inadequate cementing technique, which resulted in extensive synovitis caused by bone cement. This case underscores the critical importance of proper cementing techniques during TKA to reduce the risk of revision surgery and improve long-term outcomes.

The patient involved in this case report has given written informed consent for publication of their de-identified medical record data.

## Case presentation

Background

A 61-year-old woman who walks independently without aid with underlying diabetes mellitus, hypertension, and a history of posterior spinal instrumentation and fusion of L5/S1 underwent bilateral total knee replacement 4 years ago. She had no issues with her bilateral knees initially and recovered well. However, she began experiencing persistent swelling and pain in her right knee, which significantly impacted her mobility for six months. She sought care from her primary surgeon, who initially treated her with non-steroidal anti-inflammatory drugs (NSAIDs) for symptomatic relief. However, as the symptoms and effusion persisted, a knee joint aspiration was performed by her primary surgeon to rule out infection. Results from the aspiration showed no evidence of infection or inflammatory arthritis.

She presented to us at the orthopedic outpatient clinic seeking a second opinion as her symptoms were not getting better. Notably, there was no history of trauma or falls. While she remained ambulatory, she required a walking stick for distances greater than 50 meters. She described the pain as being more pronounced along the medial and lateral joint lines, with no radiation. It was exacerbated with walking and got better with rest. She denied any associated lower back pain, numbness, or hip pain. Additionally, there was no family history of inflammatory arthritis.

On examination, her right knee had significant effusion with fullness in both the medial and lateral gutters. The knee was mildly warm to touch but exhibited no erythema or signs of cellulitis. A positive patella tap was noted. The collateral ligaments were intact; however, a valgus stress test revealed mild laxity. The range of motion of the right knee was from 15° to 110°, but she reported pain when flexing. Patella tracking remained good, and no patella clunk was detected.

Laboratory investigations were unremarkable for infection, with a normal total white blood cell count, C-reactive protein, and procalcitonin. However, her diabetes control was sub-optimal, with a glycated hemoglobin (HbA1c) of 8.4% (Table 1).

**Table 1:** Relevant laboratory investigations HbA1c: glycated hemoglobin

Laboratory Investigation	Value (Normal value range)
Total white blood cells	6.6x10^9^/L (4.5 to 11.0 × 10^9^/L)
C-reactive protein	0.8 mg/L (< 3 mg/L)
Procalcitonin	< 0.04 ng/mL (< 0.04 ng/mL)
HbA1c	8.4% (< 5.7%)

**Figure 1:**
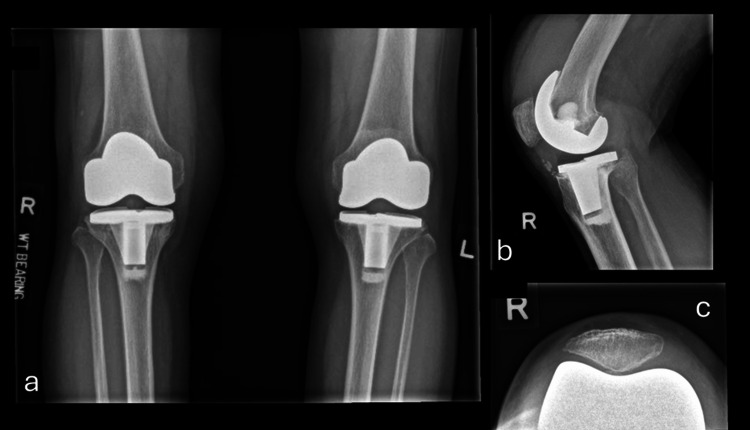
Knee radiograph during initial presentation. (a) Anteroposterior view bilateral knee; (b) Lateral view right knee; (c) Skyline view right knee.

Initial radiographs revealed loosening of the right knee implant, most notable in the tibial component, with visible bone cement fragments anterior to the tibial tray and evidence of subsidence on the lateral aspect (Figure 1).

**Figure 2:**
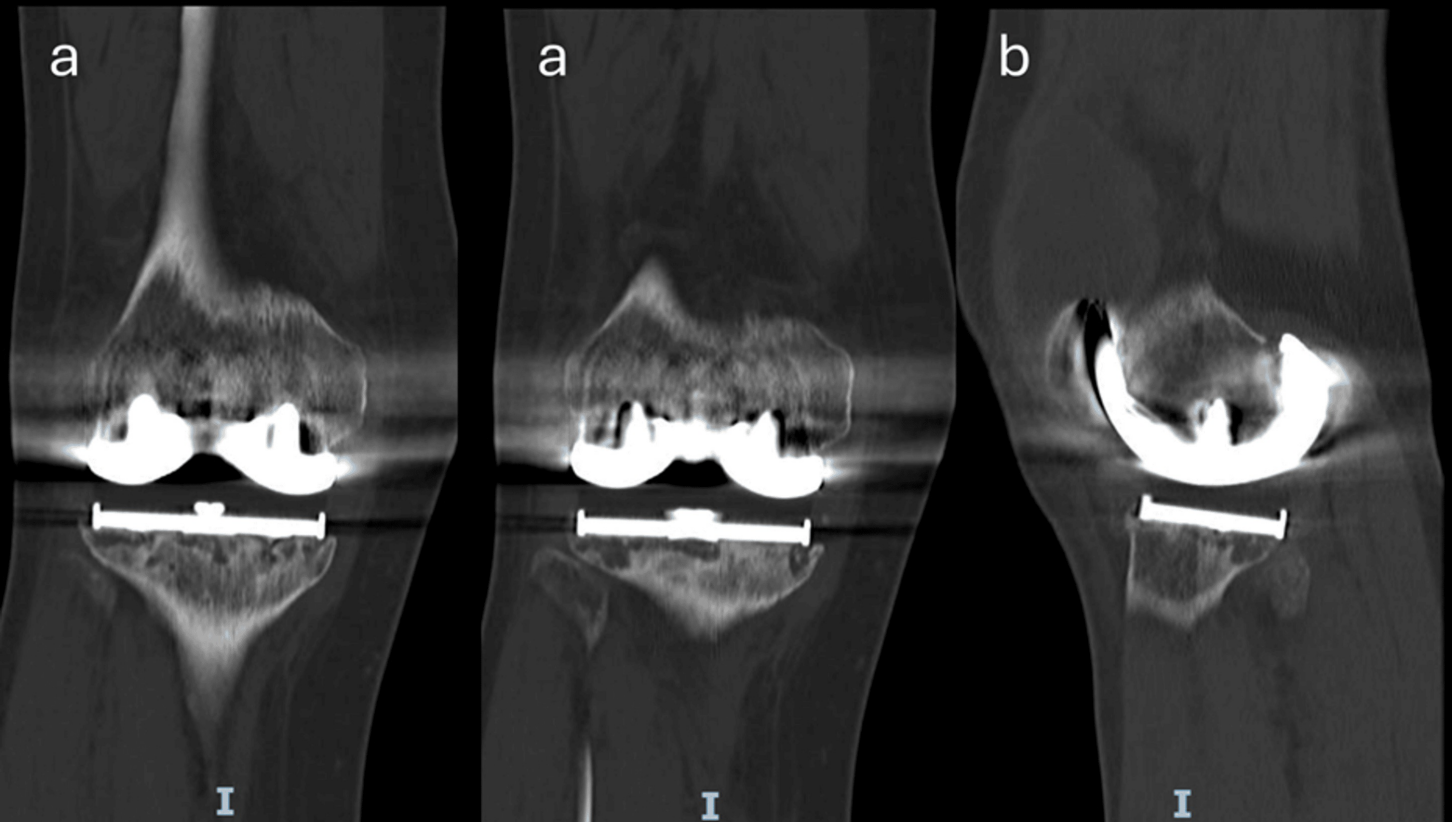
Computed tomography (CT) views of the right knee demonstrating lucency around the tibial tray. (a) Coronal views showing lucency at the tibial tray; (b) Sagittal views demonstrating lucency at the posterior aspect of the tibia tray

A computed tomography (CT) scan of the patient’s right knee was performed, revealing radiolucency around the tibial component measuring over 2 mm in thickness. These radiolucencies were irregular and ill-defined in configuration, and predominantly located posteriorly, as well as along the medial and posterolateral aspects of the tibial plateau (Figure 2). The femoral component appeared largely intact, with no evidence of gross loosening. The patient was subsequently diagnosed with aseptic loosening of the right knee, counseled regarding her condition, and proceeded to undergo a revision right TKA.

Operative details

**Figure 3:**
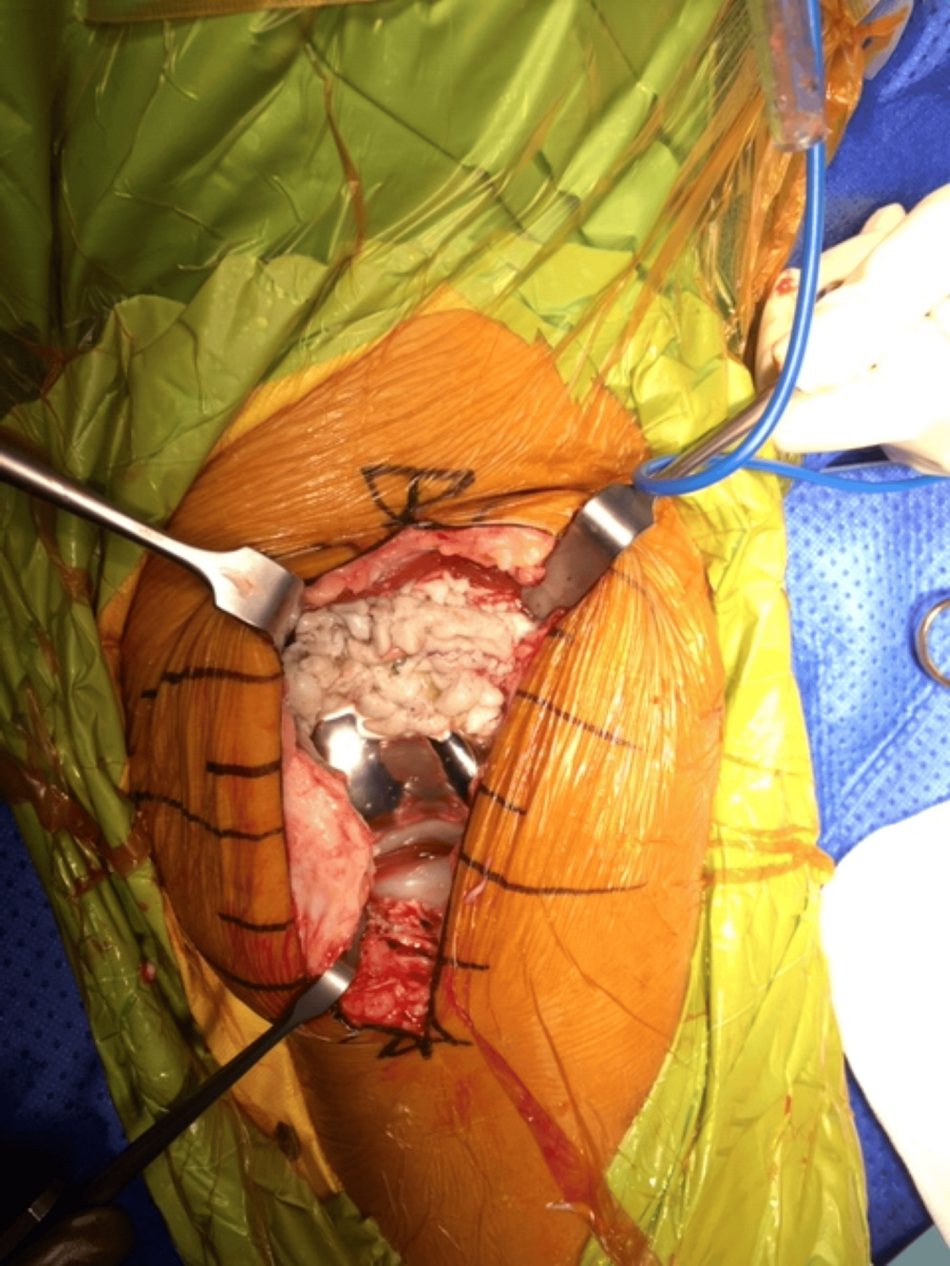
Green-hued synovitis and cementosis visualized at the distal femur.

The patient was positioned supine as a standard total knee setup. A mid-vastus approach to the knee was utilized as it is the preferred approach of the primary surgeon. When capsulotomy was performed, the synovial fluid was sent for an alpha defensin test to rule out any chance of ongoing infection. It was negative. She had extensive green-hued synovitis and bone cementosis of the distal femur with loose cement particulates as well (Figure 3). 

**Figure 4:**
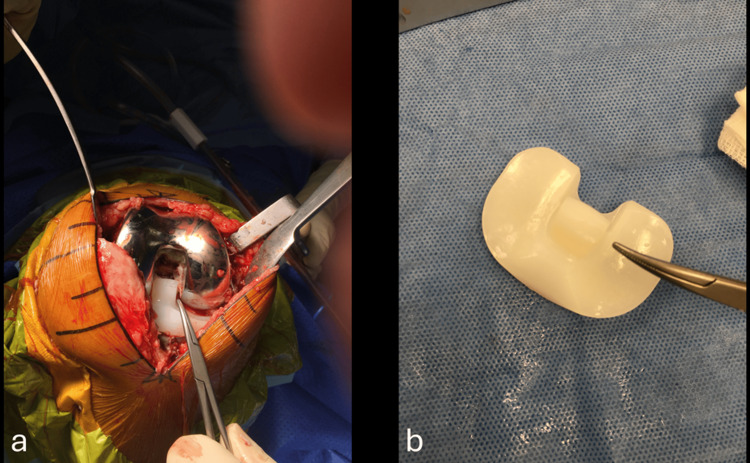
Intra-operative polyethylene liner images. (a) Excessive cement in the femoral notch; (b) Worn-out liner pole caused by repetitive friction from cement fragments.

The green coloration of the synovitis and cementosis was likely due to the use of PALACOS® cement (Heraeus Medical, Yardley, USA) by the primary surgeon, which contains a chlorophyll dye to enhance visualization during surgery. Additionally, excessive cement was identified in the femoral notch, leading to wear of the liner pole (Figure 4a, 4b).

**Figure 5:**
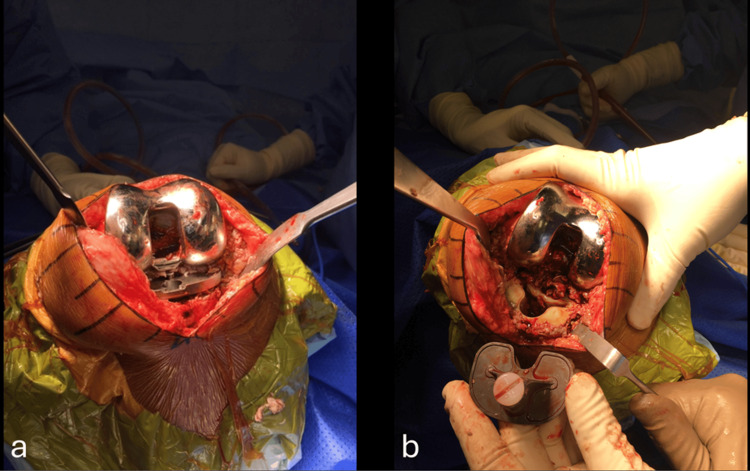
(a) Tibial tray loosening and (b) absence of cement on the tibial tray.

An extensive synovectomy was performed. Following the synovectomy, the femoral component was assessed and found to be stable, with no signs of loosening. However, aseptic loosening of the tibial tray was observed at the implant-cement interface, with no cement adhered to the tibial tray; all cement was seated on the proximal tibial cut (Figure 5a, 5b). 

The existing tibial tray was removed and revised using a stem-augmented tibial tray. A routine layered closure was then performed.

Multiple intraoperative cultures from synovial tissue and fluid were collected, all of which were unremarkable. 

Histopathological analysis of the synovial tissue suggested findings consistent with proliferative synovitis. There were florid hyperplastic/reactive features with minute fragments of refractile, non-polarisable, largely colourless material widely distributed throughout viable tissue.

Postoperative care

**Figure 6:**
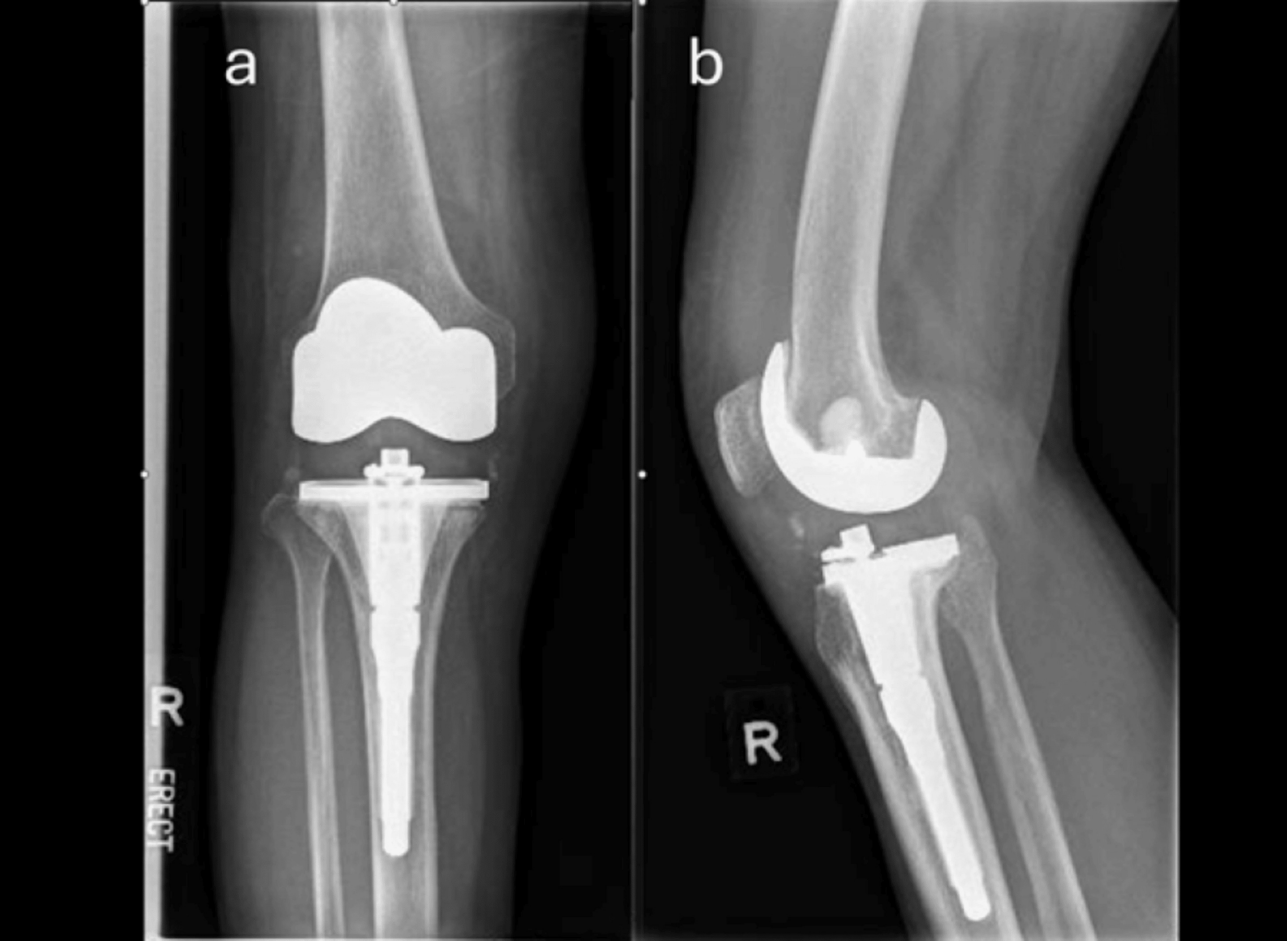
Postoperative knee radiograph. (a) Antero-posterior view; (b) Lateral view

Postoperative radiographs demonstrated satisfactory implant position (Figure 6). 

The patient was referred to the physiotherapist on postoperative day 1, and routine rehabilitative exercises were planned. She could fully bear weight on the right leg as soon as day 1 after surgery with a walking frame, and she was discharged home on postoperative day 3.

During her outpatient follow-up reviews, there were no wound-related complications. At her 2-month follow-up mark, the range of motion (ROM) of her right knee was 10° to 130°, and it was painless. She continued to experience occasional effusion, though it significantly improved compared to before surgery.

At the time of submission of this case report, she has been reviewed up to 5 years after the revision surgery. She continues to do well, with a painless, stable knee that enables her to maintain a good quality of life. She no longer experiences knee effusion or swelling and is satisfied with the outcome of her right knee.

## Discussion

TKA is one of the most performed joint replacement surgeries because it has consistently demonstrated reliability in treating knee arthritis. It boasts a reported survival rate exceeding 90% at both 10 and 15 years, with excellent patient outcomes [12,13]. However, as the number of TKA procedures increases, so do revision rates. Implant failure or pain can arise from various factors, including implant wear (with or without aseptic loosening), infection, mechanical failure, soft tissue imbalance causing instability, and uneven component wear [14].

AL remains the leading cause of revision TKA. Sharkey et al. reported an incidence of 39.9% among all TKA cases requiring revision [3]. Similarly, the Australian and United Kingdom joint registries identify aseptic loosening as the most common indication for revision, accounting for 25.3% and 32.7% of all revision TKA cases, respectively [15,16]. Several factors could contribute to AL, including implant-related factors (such as fixation type, implant design, use of constraint, and wear debris from metal, cement, or polyethylene), surgical factors (joint malalignment, ligament imbalance, and suboptimal surgical or cementing technique), and patient-related factors (such as diabetes, osteoporosis, stress shielding, and high BMI) [17,18].

In the case described above, several contributing factors to AL were identified. High BMI and diabetes are well-established risk factors for implant failure. High BMI increases mechanical stress on the prosthesis, while diabetes contributes to impaired bone quality and delayed healing, both of which increase susceptibility to AL [19,20]. Additionally, intraoperative findings of excessive cement in the femoral notch and significant polyethylene wear suggest suboptimal cementing technique and implant malalignment, further compounding the risk of AL.

The pathophysiology of AL is largely driven by particulate wear-induced osteolysis. Wear debris from polyethylene, metal, or cement generates an inflammatory response, activating macrophages and osteoclasts that promote bone resorption at the implant-bone interface. Schmalzried et al.’s concept of the “effective joint space” illustrates how debris dissemination and joint fluid dynamics can exacerbate this process, creating a self-perpetuating cycle of bone loss and implant loosening [21]. In this case, the large volume of cement debris and wear particles identified intraoperatively further corroborates this mechanism.

Optimal cementing technique is critical to the long-term stability of TKA implants. Walker et al. demonstrated that a cement penetration depth of 3 to 4 mm into the trabecular bone provides ideal fixation [22]. Proper preparation of the bone surface, including thorough pulse lavage, is essential to achieving uniform cement penetration [23]. Inadequate cementing, as observed in this case, can result in an incomplete bond between the implant and bone, leaving the construct vulnerable to early loosening.

The choice of cement viscosity also plays a role. While high-viscosity cement (HVC) allows for longer working time and easier manipulation, it may lack the adhesive properties of low-viscosity cement (LVC) during the early polymerization phase, potentially leading to weaker fixation [24,25]. Regardless of the cement type, careful pressurization and proper alignment of components are necessary to minimize the risk of future failure [26].

Management of AL involves revision surgery, with a focus on addressing both the mechanical and biological causes of failure. In this case, revision surgery was necessary to remove the failed components, debride osteolytic and inflamed synovial tissue, and achieve a stable construct. Advances in implant design, such as the use of highly cross-linked polyethylene and porous-coated components, have been developed to reduce wear and improve long-term outcomes [27]. However, patient-related factors, including BMI and comorbidities, must also be optimized to ensure the success of revision surgery.

This case emphasizes the importance of a multifaceted approach to TKA. Meticulous surgical techniques, appropriate implant selection, and management of patient-specific risk factors are critical to minimizing the risk of AL. Further research into novel materials and surgical strategies is needed to improve outcomes and reduce the burden of revision TKA in this growing patient population.

## Conclusions

This case highlights the multifactorial nature of AL in TKA and underscores the importance of optimizing patient-related factors and surgical technique to improve outcomes. The combination of patient-specific risks, such as high BMI and diabetes, along with technical issues, including suboptimal cementing and excessive debris, likely contributed to the failure in this case. Thorough preoperative planning, meticulous bone preparation, and adherence to evidence-based cementing techniques are essential to achieving long-term implant stability. This case serves as a reminder of the importance of a comprehensive approach to prevent implant failure and ensure good outcomes in TKA patients.
